# Let Bilateral Condylar Fracture Fixation Be the Norm and Not a Choice

**DOI:** 10.7759/cureus.25492

**Published:** 2022-05-30

**Authors:** Rohit Punga, Shivangi Gaur

**Affiliations:** 1 Oral and Maxillofacial Surgery, School of Dental Sciences, Sharda University, Greater Noida, IND

**Keywords:** maxillofacial trauma, sub condylar osteotomy, open reduction and internal fixation, bilateral mandibular condylar fracture, mandibular fracture

## Abstract

In lieu of a lack of consensus among the global community, it becomes imperative to address the pitfalls in the surgical management of bilateral mandibular condylar fractures with the contemporary technique of open reduction and internal fixation of only one side. This dictum inevitably leads to postoperative complications despite the best efforts of the surgical team to optimize care. We present a case series comprising residual deformities and complications arising out of the inadequate treatment of bilateral condylar fractures. We aim to highlight the significance of open reduction and internal fixation of bilateral mandibular condylar fractures and reiterate that conventional subcondylar osteotomy is an effective method for revision surgery.

## Introduction

Condylar fractures may be isolated unilateral or bilateral fractures or could present concomitantly with other maxillofacial injuries [1]. Approximately 40% to 50 % of condylar fractures are bilateral [2]. Despite this high prevalence, the management is controversial at best due to different schools of thought and a lack of consensus among surgeons worldwide. The management of bilateral mandibular condylar fractures is far more challenging as compared to unilateral fractures. The surgical community is divided over closed reduction (MMF) and open reduction and internal fixation (ORIF) of bilateral mandibular condylar fractures. Postoperative complications are more profound after bilateral mandibular condylar fractures secondary to a lack of craniomandibular articulations. Literature evidence states that 10% of bilateral mandibular condylar fractures treated conservatively need orthognathic correction later in life [3]. The practice of ORIF of one side in bilateral condylar mandibular fractures has been associated with chronic pain, malocclusion, limited mouth opening, facial asymmetry, and temporomandibular joint (TMJ) ankylosis [4]. Malocclusion secondary to conservative treatment is a sum total of the reduced ramal height bilaterally, as well as downward rotation of the mandible due to the action of suprahyoid muscles [5]. With the aid of this article, we attempt at highlighting the significance of bilateral open reduction and internal fixation of condylar fractures by presenting a case series of residual deformities and complications following unilateral open reduction and internal fixation of bilateral condylar fractures and a review of relevant literature.

## Case presentation

Case 1

**Figure 1:**
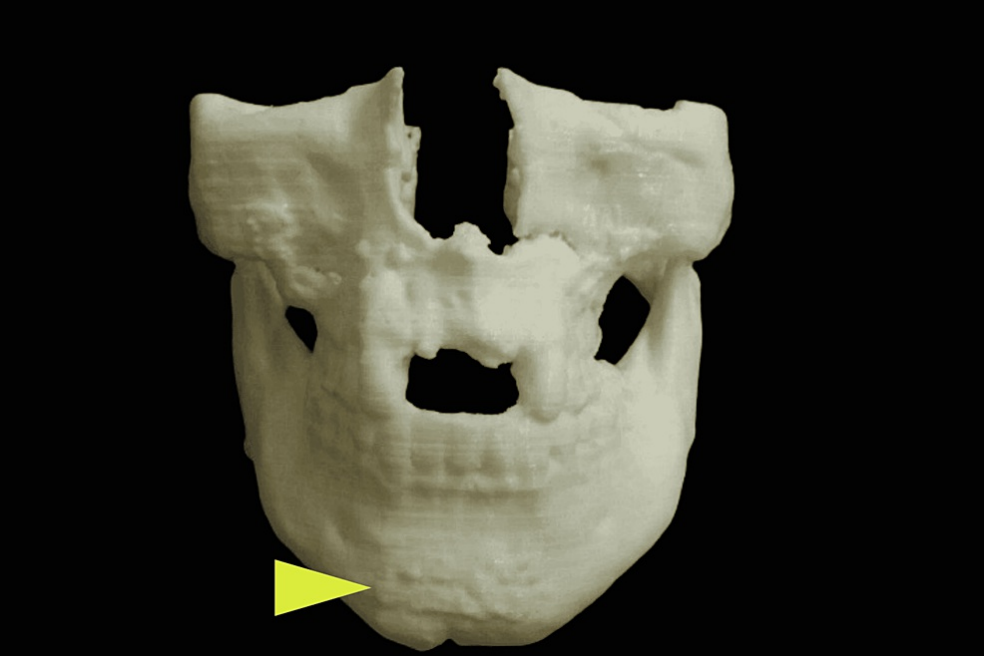
A stereolithographic model used to analyze and plan treatment, showing miniplate osteosynthesis at the mandibular symphysis region (arrow)

A 24-year-old male reported with malocclusion and widening of the lower third of the face six months after being operated on for bilateral mandibular condylar, mandibular symphysis, and bilateral Léfort I fractures at a different center. On extraoral examination, deviation of the face was noted toward the left side due to reduced posterior vertical facial height. On intraoral examination, 11, 12, 21, 22, and 31 were found to be missing. However, the edentulous space in relation to 31 was missing, but the patient confirmed its presence prior to trauma. An edge-to-edge bite was noted on the right side, whereas a scissor bite was noted on the left side, indicating a widening of the posterior mandible. CT scans revealed plate osteosynthesis in relation to mandibular parasymphysis and maxillary fractures, missing 11, 12, 21, and 22, and malunited condyles bilaterally. The patient’s preoperative and postoperative photographs were compared to estimate the extent of widening at the gonial angles and facial asymmetry. For treatment planning, 3-D stereolithographic models were obtained, which revealed collapsed mandibular symphysis region (Figure 1).

**Figure 2:**
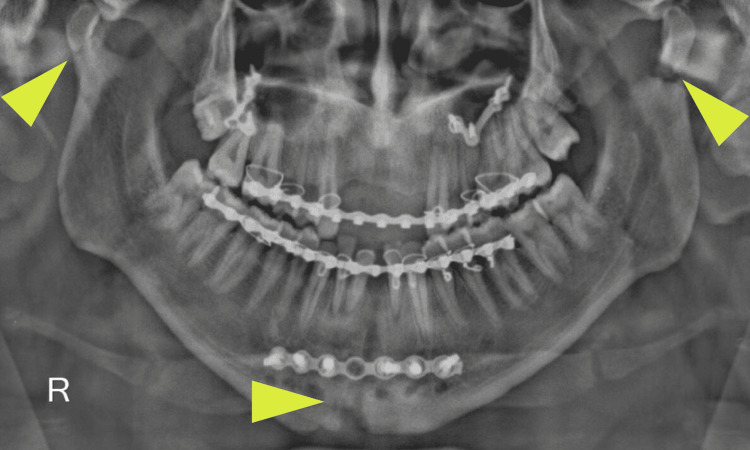
A postoperative orthopantomogram revealing bilateral mandibular subcondylar osteotomy (arrow) and vertical symphyseal osteotomy (arrows) for correction of post-traumatic deformity

The goals of the treatment were to release the bilateral condylar malunion and realign osteotomized segments to achieve facial aesthetics followed by dental rehabilitation. Intraoperatively, mandibular hardware was removed followed by bilateral subcondylar osteotomy. Fixation of condyles was not done due to a lack of reference for the pre-trauma condylar position. Vertical symphyseal osteotomy was performed to create a gap for missing 31, which helped to achieve the pre-trauma width of the lower third of the face (Figure 2).

**Figure 3:**
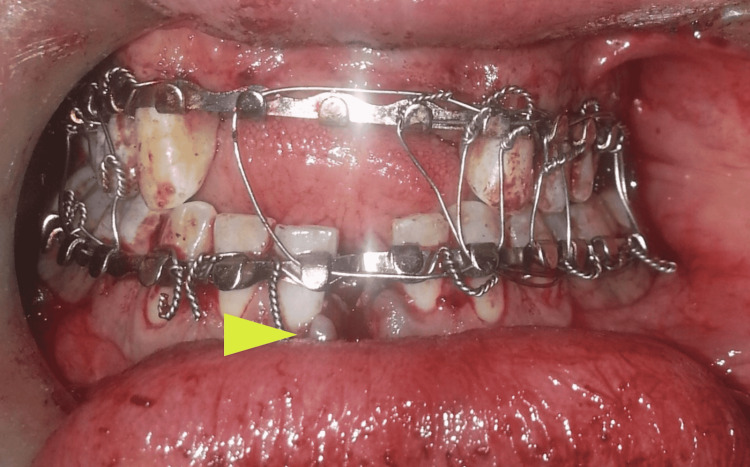
Maxillomandibular fixation was done to achieve appropriate occlusion after the creation of an edentulous space for the missing lower left central incisor (arrow) using vertical symphyseal osteotomy

Maxillomandibular fixation (MMF) was done to obtain occlusion followed by fixation of symphyseal osteotomy using the Ellis maneuver (Figure 3).

**Figure 4:**
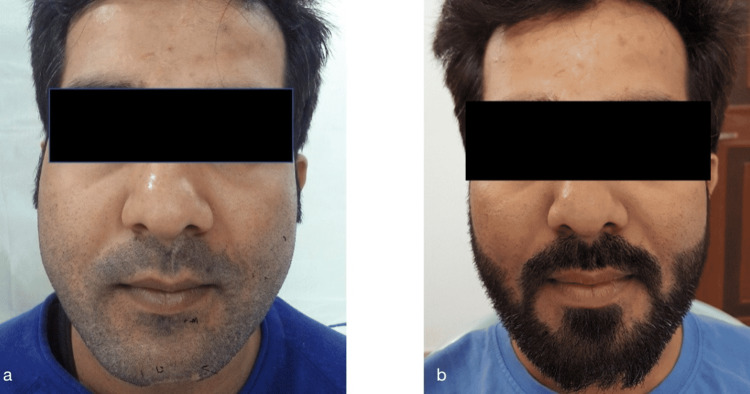
Preoperative facial profile (a) and postoperative facial profile (b)

In this case, the vertical facial height was maintained and the widening of the gonial angles was reduced thus restoring the pre-trauma facial form, which continued to be stable at the six months follow-up (Figures 4a-4b).

Case 2

**Figure 5:**
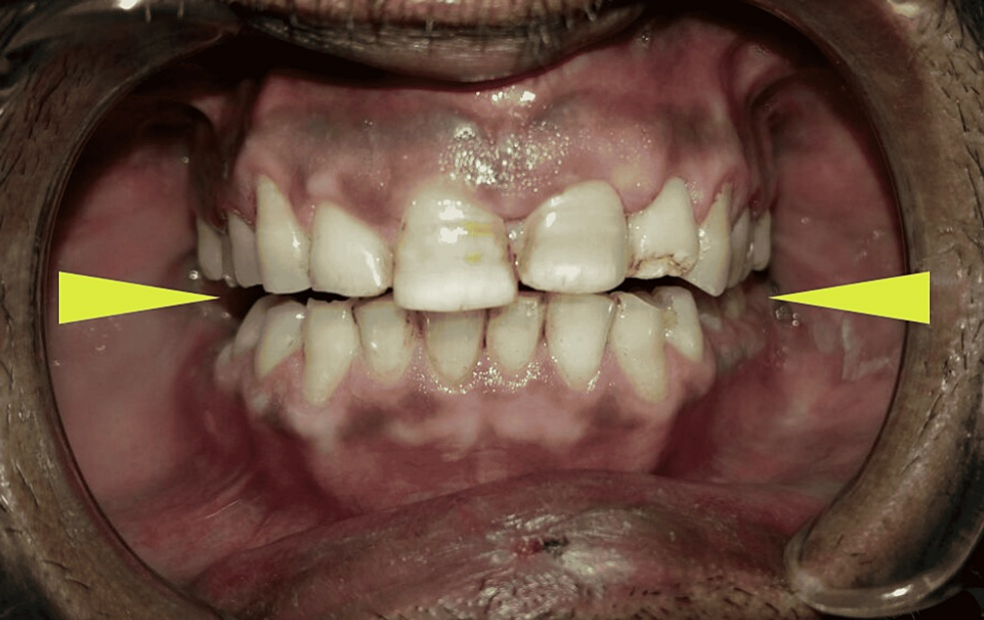
Bilateral posterior apertognathia (arrows) due to malunited bilateral mandibular condyles

A 32-year-old male reported with an alleged history of a fall from stairs one year ago and the inability to chew properly since then. On extraoral examination, increased lower facial height was observed and the intraoral examination revealed apertognathia (Figure 5).

**Figure 6:**
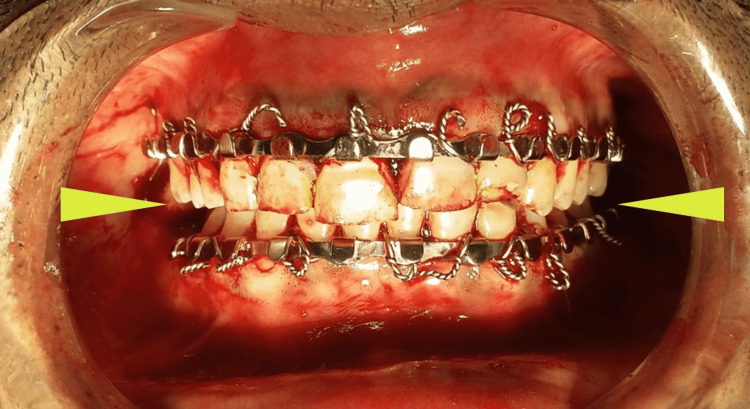
Postoperative occlusion exhibiting closure of the bilateral posterior apertognathia (arrows)

Radiographs revealed malunited bilateral mandibular condylar fractures. Bilateral subcondylar osteotomy was done to release the malunion. In order to prevent anticlockwise rotation of the mandible following the osteotomy, abnormal lateral pterygoid muscle attachments were also released. ORIF was not done because the malunited condyles had lost their normal anatomy, and it was also difficult to retrieve the bony fragments post-osteotomy, thus it was decided to maintain the patient’s normal occlusion using MMF postoperatively and let the condyles settle accordingly (Figure 6).

MMF was done for a period of three weeks postoperatively and appropriate occlusion was present at the six months follow-up. The patient was content, as his chief complaint of inability to chew food was satisfactorily addressed.

Case 3

**Figure 7:**
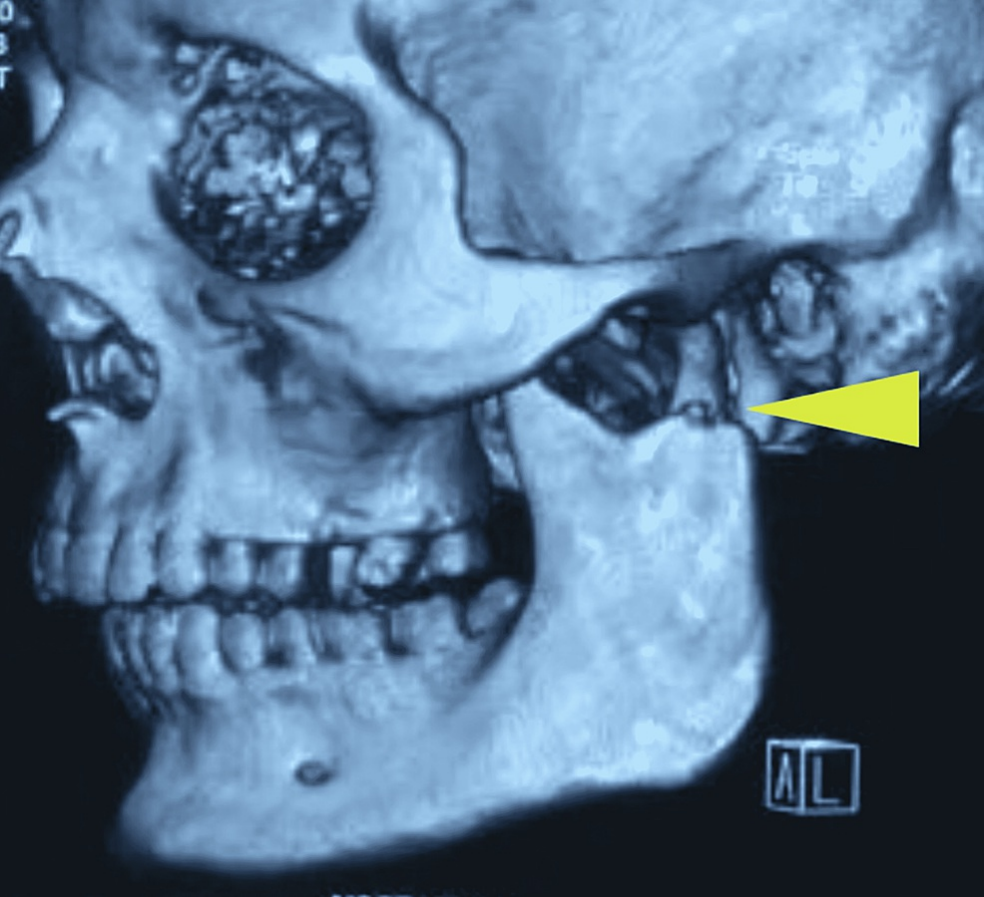
Three-dimensional computed tomography scan revealing medial dislocation of the left condylar segment (arrow)

**Figure 8:**
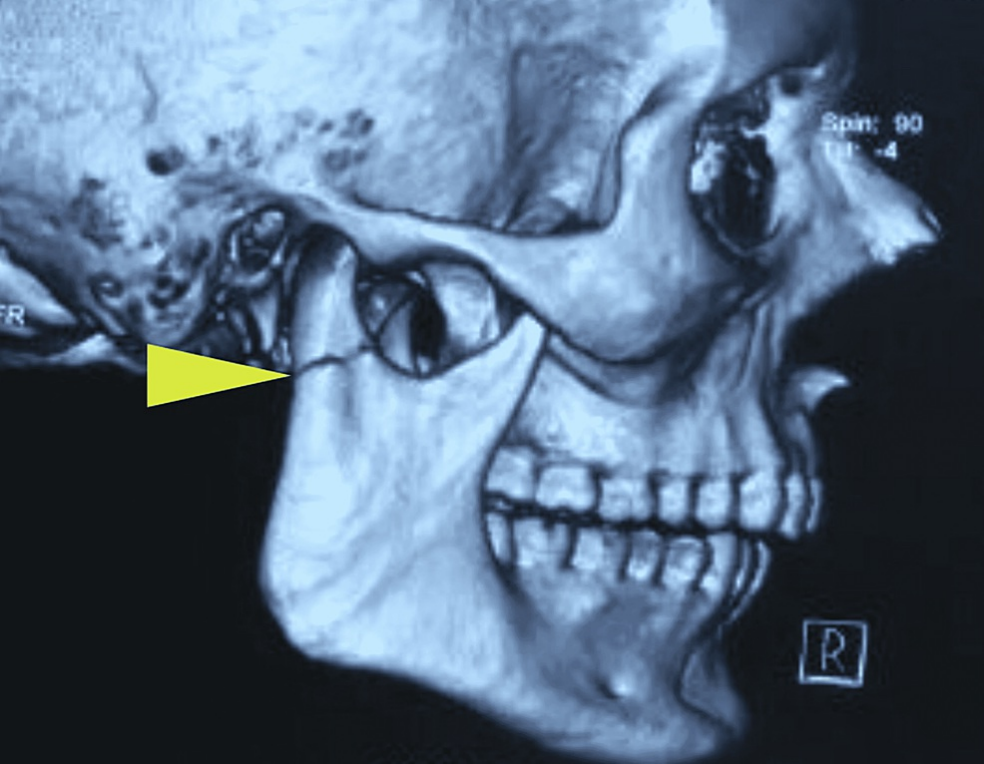
Three-dimensional computed tomography scan revealing a slightly deviated right condylar fragment (arrow)

A 32-year-old male presented with an alleged history of a road traffic accident. CT scans revealed an oblique symphysis fracture, a dislocated left condyle (Figure 7), and a slightly deviated right condyle (Figure 8).

**Figure 9:**
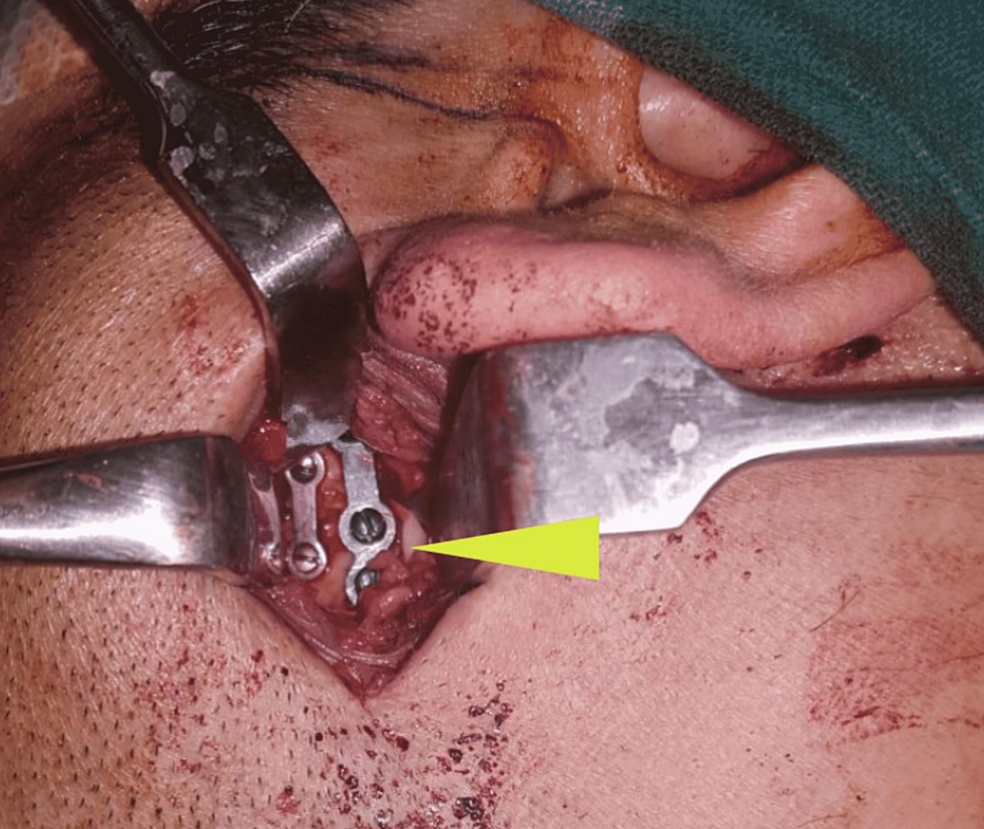
Open reduction and internal fixation of the left condylar fracture using miniplate osteosynthesis (arrow)

Telescoping of the left ramal fragment was seen, making it difficult to achieve the normal anatomic relationship of the ramal condylar unit with closed reduction. Taking into consideration the 1983 Mathes criteria, that is, fixation of the condylar fracture when the fragment angulation is more than 30°and the bone gap more than 4-5 mm, it was decided to perform ORIF for symphysis as well as the left condylar fracture (Figure 9).

MMF was kept in situ for three weeks postoperatively. Postoperatively, satisfactory occlusion, adequate vertical facial height, and acceptable function were noted, which were stable at the six months follow-up. Due to the decision to perform unilateral ORIF, the amount of hardware used was less. However, MMF could have been completely avoided if ORIF would have been performed on the right side thereby enabling early function and no post-operative morbidity pertaining to MMF.

Case 4

**Figure 10:**
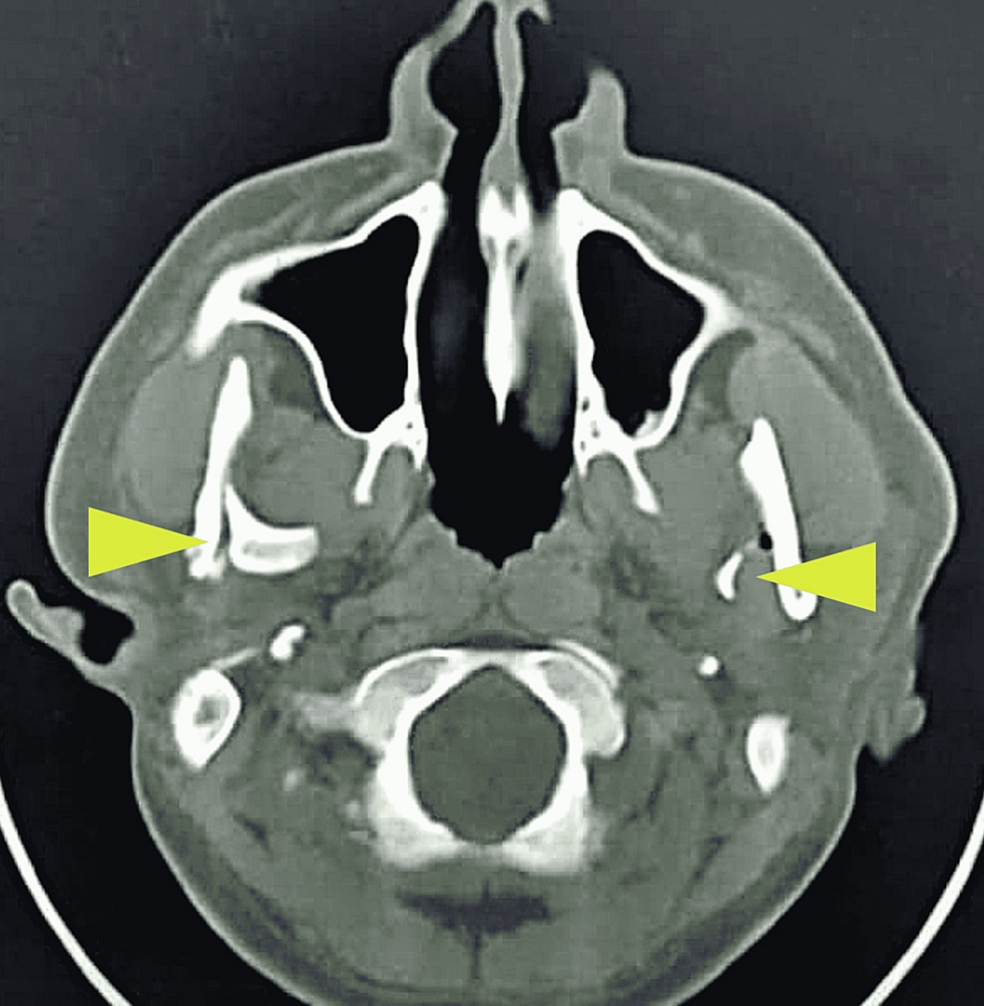
Axial section of a computed tomography scan revealing bilateral medial dislocation of mandibular condyles (arrow)

A 34-year-old male presented with a bilateral mandibular condylar neck fracture as seen on CT scan (Figure 10).

**Figure 11:**
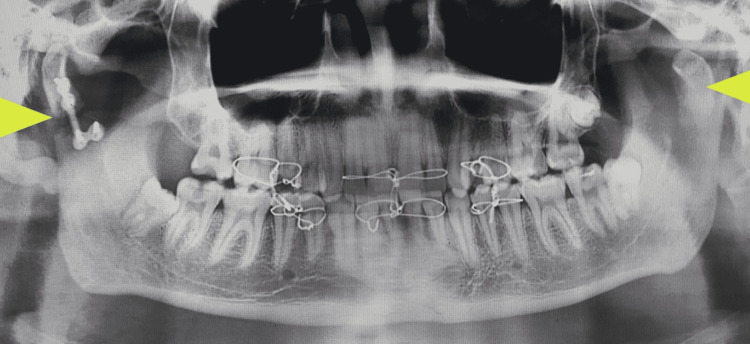
Postoperative orthopantomogram showing miniplate osteosynthesis of the right condylar fracture (arrow) and medially dislocated left condylar segment (arrow)

The fracture line in relation to the left condylar neck was relatively higher, making it difficult to retrieve the condylar fragment for ORIF. Keeping in mind the concept of fixing at least one condyle, only the right side was subjected to ORIF. An L-shaped 2 mm titanium miniplate was used to fix the right condylar neck fracture given the amount of bone available for fixation (Figure 11).

After achieving appropriate occlusion, the patient was put on MMF for a period of three weeks. After the MMF was removed, the patient presented with difficulty in opening their mouth. Since the left condyle was left unfixed, it did not attain the normal anatomic position. This, in turn, leads to inadequate mouth opening due to obstruction in temporomandibular translational motion, thus dictating the need for fixing both condyles in a case of bilateral mandibular condylar fracture.

Case 5

**Figure 12:**
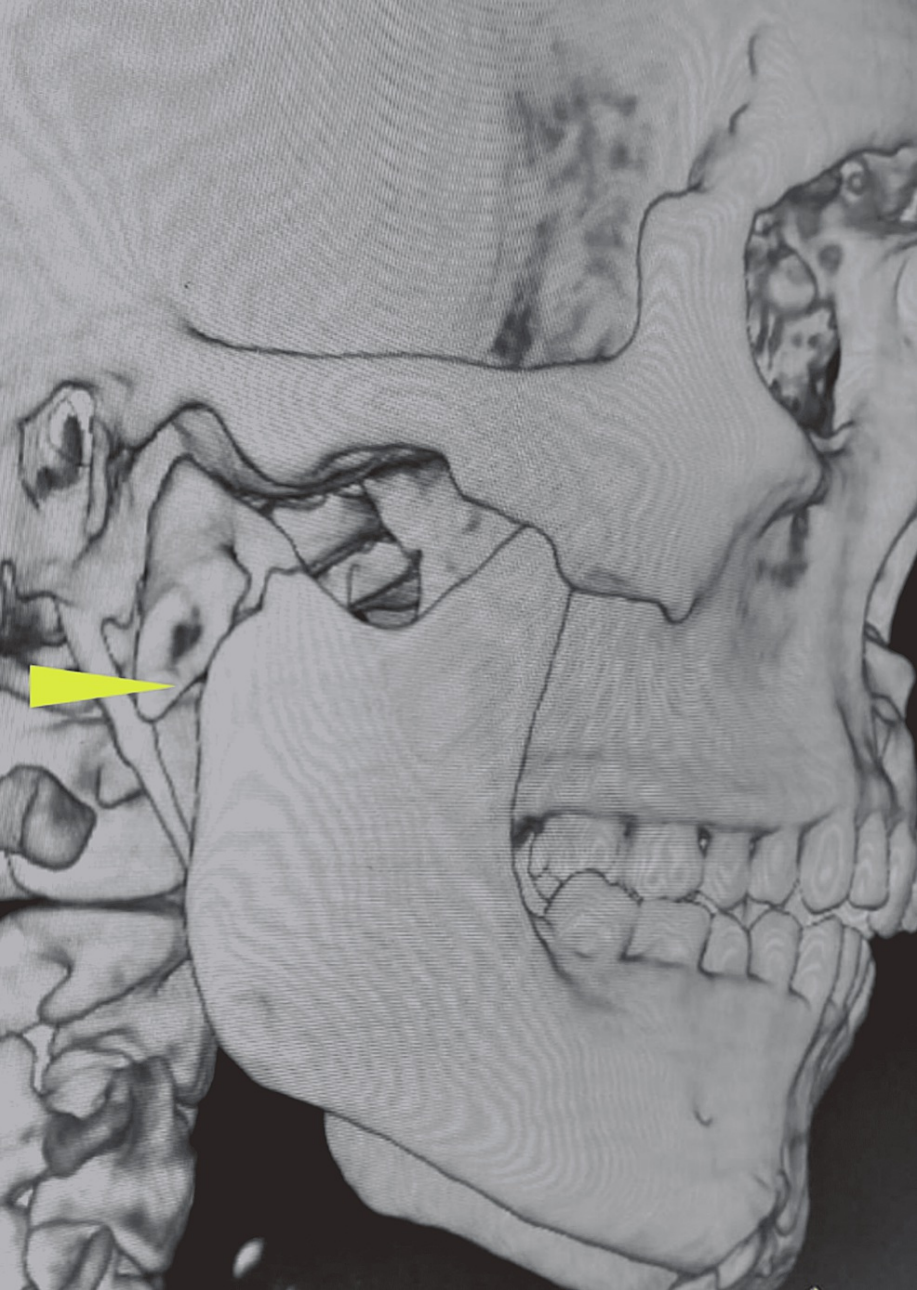
Three-dimensional computed tomography scan revealing a medially dislocated right condylar fragment (arrow)

**Figure 13:**
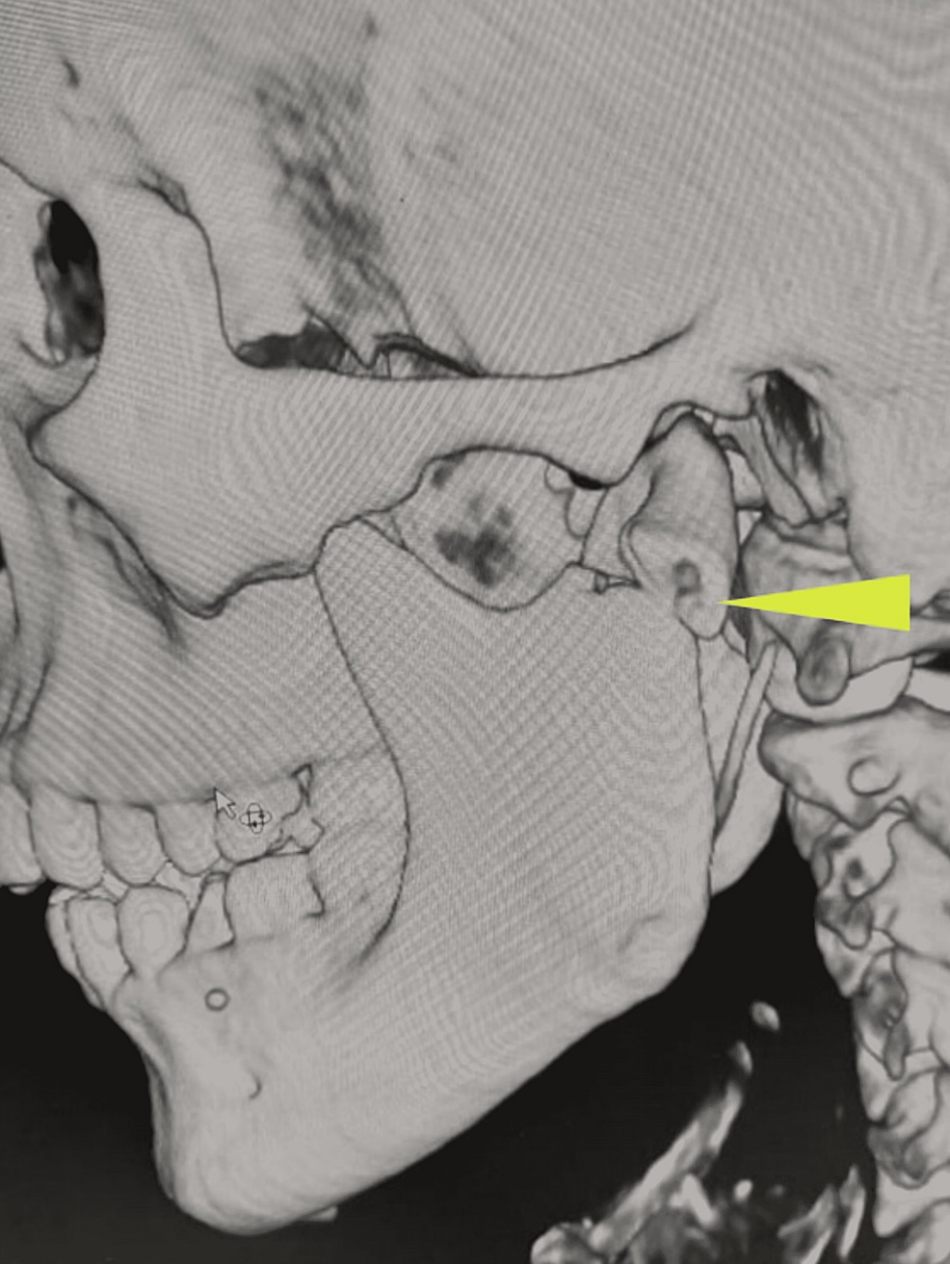
Three-dimensional computed tomography scan revealing a medially dislocated left mandibular condylar fragment (arrow)

A 28-year-old male presented with an inability to close his mouth following a road traffic accident. The CT scan revealed a dislocated bilateral mandibular condylar neck and comminuted right parasymphysis fractures (Figures 12-13).

**Figure 14:**
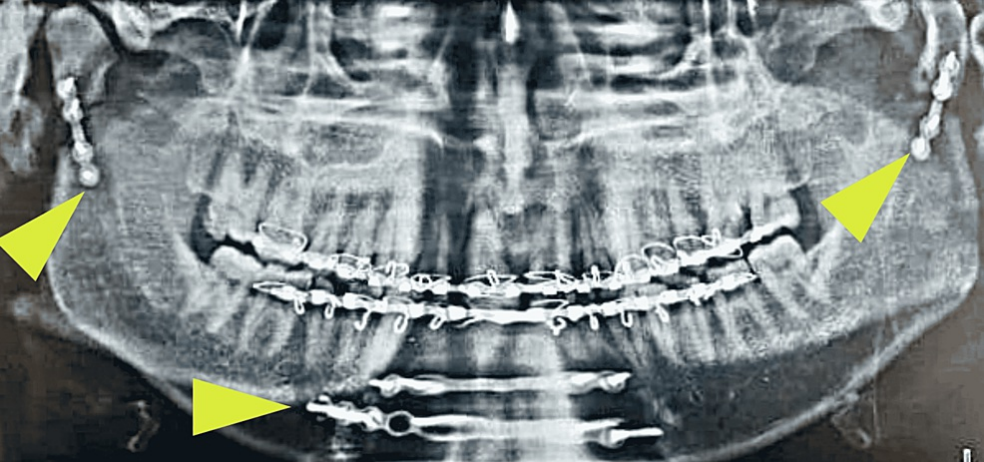
Postoperative orthopantomogram revealing miniplate osteosynthesis at the bilateral mandibular condyles and right parasymphysis region (arrows)

MMF was done preoperatively using Erich’s arch bar and guiding elastics. Intraoperatively, telescoping of both the ramal fragments was noted with the condyle dislocated medially and inverted on the right side. Both condylar fragments were reduced and fixed using a 2-mm 4 hole with a gap titanium miniplate in order to bring them to their normal anatomic position within the glenoid fossa while maintaining pre-trauma vertical facial height, followed by MMF and fixation of parasymphysis fracture (Figure 14).

**Figure 15:**
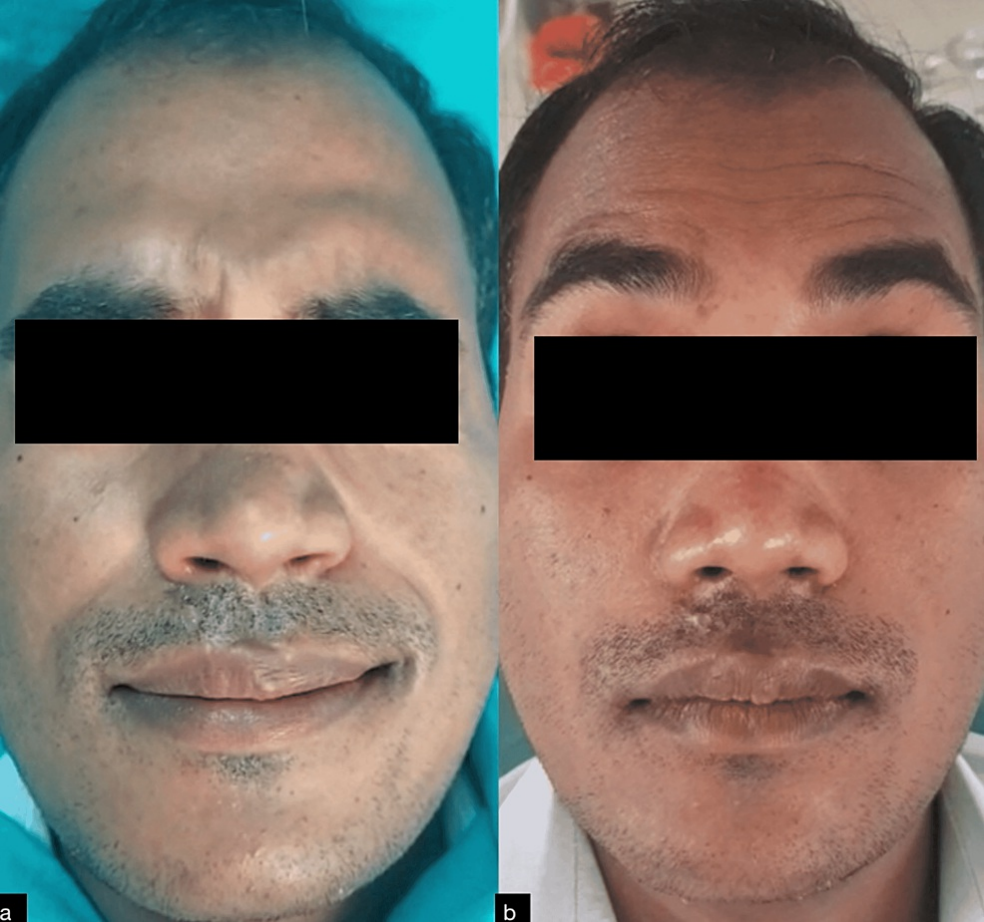
Postoperative assessment of facial nerve function revealing the satisfactory function of the temporal (a) and zygomatic (b) branches

MMF was released intraoperatively, allowing immediate return to function during the postoperative period. Appropriate vertical facial height, adequate interincisal distance, satisfactory occlusion, good facial aesthetics, and normal temporomandibular joint movements were observed two months postoperatively. Despite sustaining severe maxillofacial injuries, the present case had a better outcome, which can be attributed to early and appropriate intervention. Spending time to plan the treatment proved prudent in order to achieve perfect occlusion, adequate mandibular range of motion, and facial nerve function, which was stable at subsequent postoperative follow-up visits (Figures 15a-15b).

## Discussion

The surgical community worldwide opines differently on the best management practice for unilateral as well as bilateral mandibular condylar fractures from time immemorial. Bilateral condylar fractures are more challenging to manage as compared to unilateral fractures since the complications are more profound after bilateral involvement due to a lack of craniomandibular articulations. In bilateral condylar fractures, the displaced or dislocated condyles result in longitudinal displacement because of spasms of the masticatory muscles. The patient usually presents with increased transverse mandibular width, decreased posterior facial height, and an anterior open bite, these further amplify the difficulty in management [6]. The treatment of condylar fractures consists of two modalities, ORIF and closed treatment. Closed reduction includes the use of MMF, orthodontic appliances, and functional therapy. The principal goals of management are the re-establishment of normal occlusion, proper function, adequate vertical facial height, and acceptable cosmesis. The management technique to achieve these goals depends upon the pattern of injury, patient’s age, surgeon’s expertise, and timing of injury while contemplating the risks and benefits of treatment modality. There are certain absolute indications for ORIF of condylar fractures as per two widely accepted criteria, that is, the Zide and Kent criteria, 1983, and the Mathes criteria, 1983; however, Valiati et al. stated that except for closed treatment being an absolute indication for pediatric patients, there are still no guidelines defined for managing mandibular condylar fractures [7-9].

The complex anatomy of TMJ and related complications, such as joint resorption, facial nerve injury, wound infection, and sialocele or salivary fistula formation are some of the concerns related to ORIF. Complications related to closed reduction are facial asymmetry, malocclusion, trismus, and TMJ-related issues; particularly in the case of bilateral condylar fractures, such patients might require revision surgeries later on [10]. Most of the literature focuses on unilateral condylar fractures, which are certainly less complicated than bilateral condylar fractures; only a few studies are oriented toward bilateral condylar fractures. Complications, as reported with bilateral condylar fractures, include chronic pain, malocclusion, limited mouth opening, facial asymmetry, and TMJ ankylosis. Malocclusion is the most common complaint, especially with MMF, contributed by reduced ramal height bilaterally, as well as downward rotation of the mandible due to traction on the suprahyoid muscles [5-6].

Wang et al., 2019, found that a Chen Type II bilateral condylar fracture, which is an asymmetric bilateral fracture with one subcondylar and a contralateral intracapsular fracture, was associated with the worst functional outcomes when compared with the other two types [11]. Marker et al. investigated 39 patients who were treated conservatively; they stated that closed treatment is an effective method for treating condylar fractures, but still, bilateral condylar fractures and dislocation of the condylar head should be treated with ORIF [12]. According to Ellis and Throckmorton, displaced bilateral condylar fractures require more neuromuscular adaptations when treated by closed reduction, posing a great challenge [13]. Newman, in his study, found that 31 patients with bilateral condylar fractures who were treated with ORIF, had a better functional outcome, particularly, in mouth opening. Newman suggested that at least one fracture in bilateral cases should be treated with ORIF, as the anatomical reduction of at least one fracture lessens the need for extensive remodeling and neuromuscular adaptation [3]. Yang et al., 2002, found satisfactory functional outcomes when both the condyles were fixed; the authors stressed early rehabilitation for adequate postoperative results [14]. According to Ellis et al., 2000, bilateral condylar fractures treated by closed reduction may develop facial asymmetry, malocclusion, and an open bite [13]. Schneider et al., 2008, suggested ORIF for condylar fractures with a deviation of 10° to 45° and a 2-mm shortening of the ascending ramus [15]. Al-Moraissi and Ellis, 2015, in their systematic review and meta-analysis of 23 studies, concluded that ORIF provides superior functional clinical outcomes than closed treatment in the management of condylar fractures. Bilateral fractures increase the probability of failure of closed treatment and post-traumatic condylar deformity [16]. There are various reports in the literature suggesting adaptive remodeling of the fractured condyle to the shape of the glenoid fossa after MMF. Choi et al., 1996, studied 10 patients with bilateral condylar fractures who were treated conservatively. All the patients had normal jaw movements but the CT images showed displaced and dislocated condyles and their relationship with the glenoid fossa did not improve [17].

Delayed presentation of bilateral condylar fractures and improper or no treatment at all results in malocclusion, facial asymmetry, restricted jaw motion, and pain in the TMJ due to bone healing in an abnormal position. Such cases of bilateral condylar fractures are difficult to manage, as due to loss of craniomandibular articulations, there is no reference to guide for achieving pre-traumatic anatomy. These residual deformities can be treated using functional rehabilitation, orthodontics, subcondylar osteotomy, orthognathic surgery (mandibular osteotomy or Le fort I osteotomy depending upon dental midline discrepancy), and TMJ reconstruction. Spitzer et al., 1997, advocated the use of bilateral sagittal split osteotomy in patients with malunited bilateral condylar fractures [18]. Becking et al., 1998, included six patients with post-traumatic anterior open bite due to bilateral condylar process fractures, five patients underwent Le Fort I osteotomy with posterior impaction and autorotation of the mandible, in order to close the open bite, whereas one patient underwent bilateral inverted L ramal osteotomy [19]. Chen et al., 2013 studied 12 patients who presented with malocclusion and facial asymmetry after failed treatment of condylar process fractures or no treatment at all [20]. Eight patients who presented six months after trauma were treated with subcondylar osteotomy, just like our patients, whereas four patients who presented later than six months were treated with sagittal split osteotomy; similar results were noted in both the groups.

We advocate the use of subcondylar osteotomy as adequate results can be obtained while targeting the area of deformity only. Subcondylar osteotomy leads to the separation of a malunited condylar fragment from the ramal fragment and thus allows the independent lateral pterygoid muscle pull to bring about the condylar fragment in a better condyle-fossa relationship. Also, separate bilateral ramal fragments after bilateral subcondylar osteotomy allow for the transverse discrepancy to be corrected by the Ellis maneuver, as we did in our patient who presented with a widening of the lower third of the face. Due to the risk of avascular necrosis, the subcondylar osteotomy should preferably be performed at the site of fracture with minimal soft tissue stripping. After repositioning the condyle into the glenoid fossa, restoration of the posterior ramus height, and settlement of occlusion, the fragments can be fixed. In our cases, however, we did not fix the separated fragments but rather put the patient on MMF, as we did not have a reference for pre-traumatic-centric relation. Severely deformed or shortened ramus patients needing large jaw movement to attain pre-traumatic occlusion and post-traumatic ankylosis require TMJ reconstruction, either autologous or prosthetic, depending upon the indications. With the advent of stereolithography and virtual surgical planning, all these surgeries can be carried out with precision, using 3-D printed models and surgical guides.

## Conclusions

The risk of inadvertent complications associated with open reduction and internal fixation of bilateral mandibular condylar fractures often keeps the surgeons on their toes about appropriate management. However, the experience of surgeons in operating condylar fractures is usually not taken into account by any criteria. The old dictum of “familiarity breeds confidence” holds true for all surgical procedures. It is the authors' recommendation that the focus be on meticulously performing intricate surgeries in the condylar region using optical magnification, high-intensity light, and honing the skills of future maxillofacial surgeons. We recommend revising indications as well as re-emphasizing the need to strictly follow the existing criteria for open reduction and internal fixation of mandibular condyles to give the patient a better quality of life and early return to function instead of limiting intervention fearing potential complications. Since limited surgery has its own pitfalls as seen above, which can be easily prevented by simply fixing the fractured mandibular condyles bilaterally.
